# Comparison of serious adverse events posted at ClinicalTrials.gov and published in corresponding journal articles

**DOI:** 10.1186/s12916-015-0430-4

**Published:** 2015-08-14

**Authors:** Eve Tang, Philippe Ravaud, Carolina Riveros, Elodie Perrodeau, Agnes Dechartres

**Affiliations:** Columbia University, Mailman School of Public Health, New York, NY USA; Centre de Recherche Epidémiologie et Statistique, Inserm U1153, Hôpital Hôtel-Dieu, 1 place du Parvis Notre Dame, 75004 Paris, France; Université Paris Descartes – Sorbonne Paris Cité, Paris, France; Assistance Publique-Hôpitaux de Paris, Hôpital Hôtel-Dieu, Centre d’Epidémiologie Clinique, Paris, France; Cochrane France, Paris, France

**Keywords:** Randomized controlled trials, Registration, Reporting, Safety, Serious adverse events, Transparency

## Abstract

**Background:**

The reporting of serious adverse events (SAEs) in clinical trials is crucial to assess the balance between benefits and risks. For trials with serious adverse events posted at ClinicalTrials.gov, we assessed the consistency between SAEs posted at ClinicalTrials.gov and those published in corresponding journal articles.

**Methods:**

All records from ClinicalTrials.gov up to February 2014 were automatically exported in XML format. Among these, we identified all phase III or IV randomized controlled trials with at least one SAE posted. For a random sample of 300 of these trials, we searched for corresponding publications using MEDLINE via PubMed and extracted safety results from the articles.

**Results:**

Among the sample of 300 trials with SAEs posted at ClinicalTrials.gov, 78 (26 %) did not have a corresponding publication, and 20 (7 %) had a publication that did not match the ClinicalTrials.gov record. For the 202 remaining trials, 26 published articles (13 %) did not mention SAEs, 4 (2 %) reported no SAEs, and 33 (16 %) did not report the total number of SAEs per treatment group. Among the remaining 139 trials, for 44 (32 %), the number of SAEs per group published did not match those posted at ClinicalTrials.gov. For 31 trials, the number of SAEs was greater at ClinicalTrials.gov than in the published article, with a difference ≥30 % for at least one group for 21. Only 33 trials (11 %) had a publication reporting matching numbers of SAE and describing the type of SAE.

**Conclusions:**

Many trials with SAEs posted at ClinicalTrials.gov are not yet published, omit the reporting of these SAEs in corresponding publications, or report a discrepant number of SAEs as compared with ClinicalTrials.gov. These results underline the need to consult ClinicalTrials.gov for more information on serious harms.

## Background

Randomized controlled trials (RCTs) are considered the gold standard for assessing the effects of health care interventions and form the basis for treatment decision-making. Thus, all results from clinical trials should be transparent and accessible to all [1–3]. Results should report on efficacy and also on safety to provide an estimation of the balance of benefits and risks [4, 5]. In particular, serious adverse events (SAEs), defined as adverse events that result in death, require inpatient hospitalization or the prolongation of hospitalization, are life-threatening, or result in persistent or significant disability or incapacity or a congenital anomaly or birth defect [6], should always be completely reported [7]. However, inadequate and underreporting of trial results, especially safety results, is common [4, 5, 8, 9], which leads to biased evidence that can have serious consequences for patients. A notable example is the obscured reporting of cardiovascular risk with rofecoxib in the VIGOR study, in which naproxen, the control treatment, was presented as having a protective effect [10–12].

To increase transparency, the 2007 US Food and Drug Administration Amendments Act (FDAAA 801) required that, after September 2008, results from clinical trials conducted in the United States be made publicly available at ClinicalTrials.gov within 1 year of the completion of the trial [13–15]. In addition, after September 2009, the posting of adverse events was also mandatory [13]. All SAEs as well as other non-SAEs above a specified frequency threshold must be reported per group in addition to the total number of patients at risk. In a recent study focusing on reporting, we found that trial results, especially SAEs, were more completely reported at ClinicalTrials.gov than in corresponding published articles [16].

In this study, we examined the consistency between SAEs posted at ClinicalTrials.gov and those published in journals. We identified a random sample of trials with SAEs posted at ClinicalTrials.gov to assess whether these safety results were published and to compare the timing of availability of SAEs between ClinicalTrials.gov and publications as well as the number and type of SAEs posted at ClinicalTrials.gov and those published in corresponding journal articles.

## Methods

### Search for trials with SAEs posted at ClinicalTrials.gov

On February 2, 2014, we exported all records from ClinicalTrials.gov and, using R 3.1.1 [17] with the XML package, we identified all completed phase III or IV RCTs with at least one SAE posted at ClinicalTrials.gov. We excluded trials with only one group, trials with ≥4 groups, and phase I, I/II, II, and II/III trials. Of all eligible trials (n = 1580), we selected a random convenience sample of 300 trials to search for corresponding publications in journals.

### Search for corresponding publications in journals

In June 2014, one of the authors (ET) searched for corresponding publications in journals using the link to publications provided at ClinicalTrials.gov whenever possible. Also, MEDLINE was systematically searched via PubMed by using the ClinicalTrials.gov identification number (NCT number). If no publication was identified, MEDLINE was searched again by using keywords for drug names and condition studied. The articles identified by the search had to match the corresponding trial in terms of the information registered at ClinicalTrials.gov (same objective, sample size, location, responsible party, trial phase, and funding sponsor). If there were several publications for a trial, all publications that matched the time-frame indicated at ClinicalTrials.gov were selected. Trials for which the published article reported a different time-frame or number of groups were excluded.

A second reviewer (CR) checked the matching between ClinicalTrials.gov and the corresponding published articles for all trials. All disagreements were resolved by discussion between the two reviewers with the help of a third reviewer (AD) if needed.

### Data extraction

For the random sample of 300 trials with SAEs posted at ClinicalTrials.gov, we extracted the following characteristics from the records exported from ClinicalTrials.gov by using R 3.1.1 [17]:

1. General characteristics of the trial: lead sponsor, condition, and countries where the trial was conducted; primary completion date (defined as the date of the final collection of data for the primary outcome) and the date when results were first posted. This date was extracted from the archive record and differs from the date on which results were first received, which is available under Study Results at ClinicalTrials.gov. The difference between these two dates relates to ClinicalTrials.gov production and the vetting of the results by the US National Institutes of Health.
2. Design of the trial: phase III or IV trial and parallel or cross-over trial.
3. Interventions: details concerning the interventions for the experimental and control groups.
4. SAEs: total number of SAEs, number of SAEs per group, types of SAEs, number of SAEs per type per group, and number of participants at risk per group. We recorded the date when SAEs were first posted from the archive record.

For all trials with corresponding publications, the following information was collected from published articles, including online supplements:

1. General characteristics of the publication: journal of publication, first author, date of online publication, type of journal (general medical, specialty), and whether ClinicalTrials.gov NCT number was reported in the published article.
2. SAEs: whether SAEs were reported and whether they were reported by number per group. If the total number of SAEs per group was not reported, we noted whether the number of the most common SAEs (those observed above a certain frequency or threshold rate), number of SAEs related to treatment, or toxicity-graded events were reported instead. We also extracted the types of SAEs reported and noted whether the number per type and per group was reported. Again, if all types were not reported, we noted whether the types reported were common event types, those observed above a certain frequency or threshold rate, those related to treatment, or toxicity-graded events. We also collected the number of participants at risk for each group.

If several publications were identified for the same trial, we extracted safety results from all corresponding publications having the same time-frame as reported at ClinicalTrials.gov. For trials with multiple phases (e.g. lead-in or induction, double-blind randomized treatment, and follow-up or extension) reported at both ClinicalTrials.gov and in published articles, we extracted SAE data only for the double-blind randomized treatment period. For published articles reporting pooled results from multiple trials, we considered the data to be missing if we were unable to extract SAE data that corresponded to the specific trial.

All data were extracted from the published article independently by two reviewers (ET, CR), independently of data collected from ClinicalTrials.gov. One of the reviewers (CR) was blinded to the hypothesis. All disagreements were resolved by discussion to reach a consensus, including intervention by a third reviewer (AD) in case of discrepancies.

### Statistical analysis

Descriptive data are reported with numbers (percentages) and median (quartile 1–3 [Q1–3]). We compared time between primary completion date as reported at ClinicalTrials.gov and date of the SAEs first publicly posted at ClinicalTrials.gov or the date of the first online publication in journals reporting the number of SAEs per group by the Kaplan–Meier method. Trials for which the number of SAEs per group were not reported in a published article on June 2014 were censored at this date for the estimation of time between the primary completion date and online publication of SAEs. For trials with both results posted and published, we compared the number of SAEs reported at ClinicalTrials.gov and in the published article. To simplify, for three-group trials with two groups representing different doses of the same treatment, we combined these two groups. For trials comparing three different treatments, we selected the main comparison according to the ClinicalTrials.gov posting and without knowledge of the results. Analyses involved the use of R 3.1.1 [17].

## Results

**Fig. 1:**
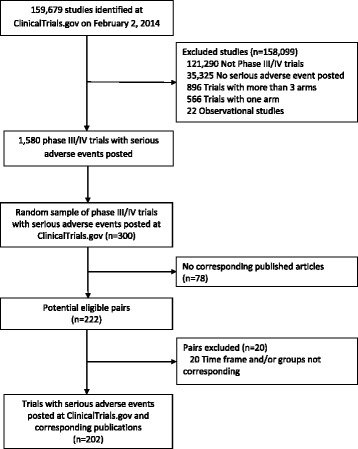
Flow of the selection of relevant trials

Figure 1 describes the selection of trials. Briefly, from the 159,679 studies recorded at ClinicalTrials.gov on February 2, 2014, there were 1,580 phase III or IV randomized drug trials with two or three groups having results posted. We selected a random sample of 300 trials to search for corresponding publications. Among the 300 trials, 78 (26 %) had no corresponding published article. From the remaining 222 trials with results both posted and published, we excluded 20 trials for which the time-frame and/or number of groups reported in the article did not match those posted at ClinicalTrials.gov. Table 1 describes the characteristics of the 300 randomly selected trials and the 202 trials with corresponding published articles.

**Table 1:** Characteristics of the random sample of 300 phase III or IV trials with at least one serious adverse event (SAE) posted at ClinicalTrials.gov for which publications were sought

Characteristic	Sample of trials with at least one SAE posted at ClinicalTrials.gov	Sample of trials with corresponding published article
	(n = 300)	(n = 202)
Study phase		
III	234 (78)	168 (83)
IV	66 (22)	34 (17)
Study design		
Parallel groups	287 (95)	197 (97)
Cross-over	9 (3)	5 (3)
Factorial	2 (1)	0 (0)
Other	2 (1)	0 (0)
No. of intervention groups		
Two	233 (78)	169 (88)
Three	60 (20)	33 (12)
Other	7 (2)	0 (0)
Primary funding source		
Industry	264 (88)	178 (88)
US National Institutes of Health	8 (3)	7 (3)
US federal funding	1 (0)	1 (1)
Other	27 (9)	16 (8)
Medical condition		
Endocrinology	41 (14)	32 (16)
Infectious diseases	38 (13)	22 (11)
Cardiology	31 (10)	18 (9)
Neurology	29 (10)	23 (11)
Oncology	29 (10)	23 (11)
Rheumatology	22 (7)	13 (7)
Pulmonary	20 (6)	15 (7)
Other	90 (30)	56 (28)
Study location		
At least one site in the United States	205 (68)	140 (69)
No site in the United States	95 (32)	62 (31)
Type of journal		
Specialty		165 (82)
General		37 (18)
ClinicalTrials.gov NCT reported in article		
Yes		162 (80)
No		40 (20)

Data are no. (%)

### Time to availability of SAEs at ClinicalTrials.gov and in published articles

**Fig. 2:**
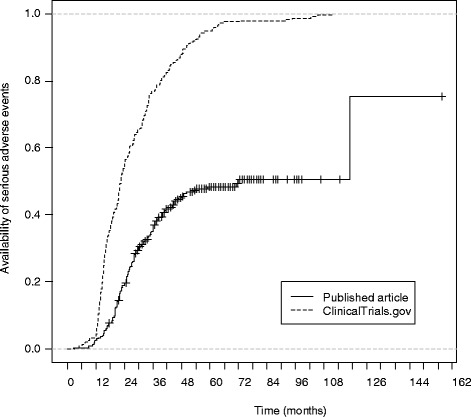
Comparison of time from primary completion date to posting of serious adverse events (SAEs) per group at ClinicalTrials.gov and to the reporting of SAEs per group in published articles

The difference between the median time to the availability of SAEs at ClinicalTrials.gov and in published articles was 50 months (95 % confidence interval [95 % CI]: 26–98 months) with the median time between primary completion of trials and SAEs publicly posted at ClinicalTrials.gov of 22 months (Q1–3, 15–35) and median time between primary completion date and availability of SAEs in published articles of 72 months (Q1–3, 27–119; Fig. 2).

### Reporting of the number of SAEs per treatment group

**Fig. 3:**
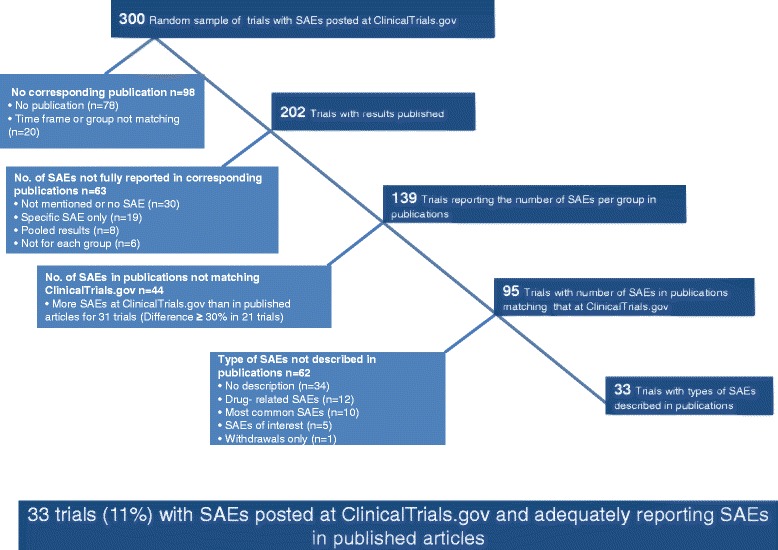
Reporting of serious adverse events (SAEs) in published articles for a random sample of 300 trials with SAEs posted at ClinicalTrials.gov

From the 202 final pairs of trials, the number of SAEs per group was reported in the published article for 139 (69 %); 26 (13 %) published articles did not mention SAEs at all and 4 (2 %) reported no SAEs. Of these 30 articles, for 12 trials, the number of SAEs posted at ClinicalTrials.gov was >10 and for 6, >100. For 33 trials, the reporting of SAEs was incomplete in the published article: 10 reports (5 %) described only drug-related SAEs or SAEs of interest and 9 (4 %) reported adverse events with grade ≥3 instead. In 8 articles, the results were pooled from several trials and we were not able to extract the number of SAEs for each individual trial. Three published articles (1 %) did not report the number of SAEs per group, and 3 (1 %) reported only SAEs for the experimental group (Fig. 3).

Among the 139 trials that reported SAEs per group in the published article, for 44 (32 %), the number of SAEs per group did not match that posted at ClinicalTrials.gov; for 32 of these, the number of SAEs was different for both groups and, for 12, only one group. For the 32 trials with number of SAEs different for both groups, 22 (69 %) had more SAEs reported at ClinicalTrials.gov than in the published articles. For 15 of these trials (68 %), the difference between the number posted at ClinicalTrials.gov and the published article was ≥30 % for at least one group. For the 12 trials where number of SAEs was different for one group only, 9 trials had more SAEs reported at ClinicalTrials.gov than in the published article. For 6 of these trials, the difference between the number posted at ClinicalTrials.gov and the published article was ≥30 %.

### Reporting of details of SAEs

Of the 95 trials with number of SAEs matching that at ClinicalTrials.gov, all types of SAEs that occurred were reported for 33 (35 %) in the corresponding published articles. In 34 published articles (36 %), the type of SAE was not reported at all. Other publications reported the types of SAEs for only drug-related SAEs (n = 12), the most common SAEs (n = 10), the SAEs of interest (n = 5), or SAEs leading to withdrawal (n = 1).

Overall, only 33 trials (11 %) out of the random sample of 300 trials with SAEs posted at ClinicalTrials.gov had a publication reporting matching SAE numbers and describing the type of SAE (Fig. 3). The characteristics of these 33 trials are presented in Table 2. In brief, 88 % of these trials were phase III trials, 82 % had a private funding source, 73 % had at least one site in the United States, and 73 % were published in a specialty journal.

**Table 2:** Characteristics of the 33 trials with adequate reporting of serious adverse events

Characteristic	n (%)
	n = 33
Study phase	
III	29 (88)
IV	4 (12)
Study design	
Parallel groups	31 (94)
Cross-over	2 (6)
No. of intervention groups	
2	30 (91)
3	3 (9)
Primary funding source	
Industry	27 (82)
US National Institutes of Health	2 (6)
US federal funding	1 (3)
Other	3 (9)
Medical condition	
Endocrinology	6 (18)
Infectious diseases	4 (12)
Cardiology	3 (9)
Gynecology	3 (9)
Neurology	3 (9)
Rheumatology	3 (9)
Psychiatry	3 (9)
Other	8 (24)
Study location	
At least one site in the United States	24 (73)
No site in the United States	9 (27)
Type of journal	
Specialty	24 (73)
General	9 (27)
ClinicalTrials.gov NCT reported in article	
Yes	27 (82)
No	6 (18)

## Discussion

Herein, we identified a random sample of trials with SAEs posted at ClinicalTrials.gov to assess whether these safety results were reported in published articles and, if yes, whether there were discrepancies between the publication and the registry data. Our results highlight that the reporting of SAEs in published articles remains a major problem. For a sample of 300 trials with SAEs posted at ClinicalTrials.gov, among 202 with a matching publication, 30 (15 %) did not mention SAEs or reported no SAEs in the corresponding publications. The number of SAEs per group was frequently not reported in the published articles and when it was reported, discrepancies with the numbers posted at ClinicalTrials.gov were common, with frequently more SAEs reported at ClinicalTrials.gov than in the published article.

Restricted space in articles is a frequently cited reason for incomplete reporting of harms [4, 18]. However, the assessment of the balance between benefits and risks should be the core of trial reports. Failure to report SAEs may lead to a biased safety profile and erroneous decision-making, with major consequences for patients. Despite the extension of the Consolidated Standards of Reporting Trials (CONSORT) statement published in 2004, which provides guidelines on reporting harms-related data [7], reporting of safety data in published articles of clinical trials continues to be suboptimal [5, 16, 19, 20], with poor adherence to the statement [21–24]. According to a recent study, only 63 % of published articles reported the total number of SAEs by group [16].

In a previous article focusing on completeness of reporting, we found that SAEs were significantly more completely reported at ClinicalTrials.gov than in the published articles (99 % vs. 63 %, *P* <0.0001) [16]. This result was particularly troubling, but one explanation could be that SAEs were not reported in published articles because there were none.

Our results identified some trials not mentioning SAEs or reporting no SAEs in the published article, despite these being reported at ClinicalTrials.gov. Furthermore, when SAEs were reported in published articles, discrepancies with the number posted at ClinicalTrials.gov were common, with frequently more SAEs reported at ClinicalTrials.gov than in the published article. Although we do not know which the ‘true’ results are, we believe that these discrepancies clearly outline problems in the reporting of SAEs. Two studies comparing results posted at ClinicalTrials.gov and in peer-reviewed publications also showed discrepancies in the number of SAEs [18, 25, 26]. The originality of our approach was the identification of trials for which we had knowledge of SAEs to assess whether and how these safety results were reported in published articles.

Our results have important implications. Our results highlight that ClinicalTrials.gov provide more information on serious harms, whereas these events are frequently underreported in published articles. For systematic reviewers, they outline the interest of using ClinicalTrials.gov to find safety results not yet published in journals and for trials with both SAEs posted and published, to compare the rate of SAEs. In case of discrepancies, we recommend systematically contacting authors for clarification and performing sensitivity analyses in case of non-response to assess to what extent these discrepancies may affect the meta-analysis result. For journals, they question the peer-review process, in that the assessment of data recorded in registries including results and harms when available should be part of the process to assess if there are any discrepancies that could bias the results. In case of discrepancies, investigators should be contacted for clarification. They also raise questions about how reporting guidelines, especially the CONSORT harms, are implemented by journals, with a need for more active endorsement. Templates with mandatory reporting of critical elements, such as that used at ClinicalTrials.gov [14], could improve the reporting of safety results in journals. For policymakers, our results advocate an extension to all countries of the mandatory posting of trial results. Besides their use for limiting publication bias and selective outcome reporting, public registries may help improve transparency of results in clinical trials. Accordingly, in April 2014, the European Union voted to adopt the Clinical Trials Regulation, which requires the registration of all clinical trials conducted in Europe and posting of trial summary results in the European Clinical trials Database (EudraCT) within 1 year after trial completion [27, 28]. Nevertheless, compliance to the legal requirement in the United States is low [16, 29–33] despite civil monetary penalties (up to $10,000 a day) and, for federally funded studies, the withholding of grant funds in cases of non-compliance [14]. Therefore, compliance must be improved. A recent article showed that sending emails to responsible parties of completed trials that do not comply with the FDAAA legal requirement to post results significantly improved the posting rate at 6 months [34].

### Limitations

We may not have identified all published articles because we searched only MEDLINE for publications. Further, for trials without publications, the results could be published at a future date because publication in journals may take time due to multiple submissions. Some trials may have multiple publications with different results reported. In this case, we did not include all reports resulting from the trial but only the reports that included safety data and matched the time frame reported at ClinicalTrials.gov. Finally, this study focused on trials assessing pharmacological treatments, but non-pharmacological treatments can also incur SAEs.

## Conclusions

Our results reveal that many trials with SAEs posted at ClinicalTrials.gov are not yet published, omit the reporting of these SAE in corresponding publications, or report a discrepant number of SAEs as compared with ClinicalTrials.gov. Consulting safety results posted at ClinicalTrials.gov, when available, is crucial for more information on serious harms.

## Abbreviations

CONSORT: Consolidated Standards of Reporting Trials
FDAAA: Food and Drug Administration Amendments Act
RCT: Randomized controlled trial
SAE: Serious adverse event
